# Between-practice variation in chronic obstructive pulmonary disease diagnosis guideline compliance: an observational study

**DOI:** 10.3399/BJGPO.2024.0263

**Published:** 2026-02-25

**Authors:** Alex Bottle, Alex Adamson, Benedict Hayhoe, Jennifer K Quint

**Affiliations:** 1 School of Public Health, Imperial College London, London, UK

**Keywords:** respiratory illness, guidelines, large database research, chronic obstructive pulmonary disease, primary health care, observational study

## Abstract

**Background:**

Early chronic obstructive pulmonary disease (COPD) diagnosis is vital, but little is known about compliance with relevant diagnostic guidelines or variation in primary care.

**Aim:**

To quantify between-practice variations in guideline compliance and over time.

**Design & setting:**

An observational study in English primary care.

**Method:**

The Clinical Practice Research Datalink was used to assess the use of four pre-diagnostic investigations (spirometry, chest X-ray, full blood count [FBC], and body mass index [BMI]) by GP practices for patients with COPD recorded first in primary care, in three time periods: 2006–2007 (cohort 1), 2016–2017 (cohort 2), and March–August 2020 (cohort 3). Multilevel logistic regression models quantified the non-random variation between GP practices in spirometry around diagnosis. Funnel plots counted the proportion of outliers.

**Results:**

Cohort totals were 31 676 (cohort 1), 37 393 (cohort 2), and 3368 (cohort 3). Overall, the mean age was 68.3 years (standard deviation 12.0), with 46.1% female. The use of pre-diagnosis spirometry improved a little in cohort 2 (74.2%) on cohort 1 (62.8%) but fell back for the COVID-19-era group (61.1%). In contrast, chest X-ray, FBC, and BMI all improved after cohort 1 and were maintained for the COVID-19 cohort; almost all patients received one of these investigations. The proportion receiving all four investigations before diagnosis jumped from 26.6% in cohort 1 to 46.7% in cohort 2 and was maintained in cohort 3 (43.0%). Modelling and funnel plots showed considerable non-random variation in spirometry use by practice, although with some improvement since cohort 1.

**Conclusion:**

The recording of spirometry and chest X-rays warrants further and consistent improvement in the context of COPD care.

## How this fits in

Early diagnosis of chronic obstructive pulmonary disease (COPD) is vital, but little is known about compliance with relevant diagnostic guidelines or variation between primary care professionals. Compliance with National Institute for Health and Care Excellence (NICE) guidelines for diagnostic tests improved between 2006–2007 and 2016–2017, and was maintained during the early COVID-19 era. There exists considerable non-random variation in spirometry use by practice, although with some improvement since 2006–2007. The recording of spirometry and chest X-rays warrants further improvement overall and more consistency between GP practices.

## Introduction

Early diagnosis of COPD is crucial, but there is limited quantification of compliance with relevant diagnostic guidelines or variation between primary care professionals and whether these have changed over time. Our work with English data on heart failure (HF), a long-term condition with comparable diagnostic and management challenges, found low compliance with NICE diagnostic guidelines and considerable variation by GP practice.^1^ There have been several relevant NICE clinical guidelines on the diagnosis of COPD. The first was published in 2004, while NG115, which is relevant to the COVID-19 era, was published in December 2018.^2^ Both recognise that there is no single diagnostic test, although spirometry is essential to assess airway obstruction, and that distinction from asthma is crucial. Both state that a COPD diagnosis should be considered in patients aged >35 years who have a risk factor (generally smoking) and who present with ≥1 of five symptoms.^2^ The guidelines recommend performing the following investigations for patients presented with suspected COPD in primary care:

1. spirometry to assess airway obstruction and assist in distinction from asthma;
2. chest radiograph (X-ray) to exclude other pathologies;
3. FBC to identify anaemia or polycythaemia; and
4. calculation of BMI.

Additional investigations, such as those related to HF, asthma medications, or respiratory or COPD referrals, are advised as optional. Compliance with guidelines for many long-term conditions, including COPD, is financially incentivised in the Quality and Outcomes Framework (QOF), a national pay-for-performance scheme in UK primary care introduced in 2004.

This study aimed to quantify the variation in NICE guideline compliance in three cohorts defined by time periods using data from a representative sample of GP practices in England.

## Method

### Data

The Clinical Practice Research Datalink (CPRD) Aurum collects anonymised patient electronic health records from GP practices using the EMIS software, representing around 13% of the population in England. It includes patient-level data on demographics, tests, symptoms, diagnoses, therapies, prescriptions, and referrals to secondary care.^3^ Data at all CPRD Aurum practices are routinely linked to Office for National Statistics (ONS) death registration data, Hospital Episode Statistics (HES) datasets covering hospital activity, and small area-level socioeconomic data (Index of Multiple Deprivation [IMD]).

### Cohort definition

To determine the first COPD record in either primary or secondary care, we obtained records for all patients aged >35 years with a diagnosis of COPD in primary or secondary care records. Each patient’s index (diagnosis) date was defined as the first record of COPD, either in primary care record data via SNOMED-CT codes or of acute exacerbations of chronic obstructive pulmonary disease (AECOPD) in hospital admission data via International Classification of Diseases, 10th Revision (ICD-10) codes J44.9 (first position), J22, and J44.9 together in first and second position, or J44.0 or J44.1 in any position algorithms.^4,5^ We included all patients with an index date between 1 January 2006 and 31 December 2007 (cohort 1) and between 1 January 2016 and 31 December 2017 (cohort 2); a smaller COVID-19-era group for March to August 2020 made up cohort 3. Finally, patient-level data were extracted based on these criteria:

- patients aged >35 years flagged as having records of acceptable data quality by CPRD;
- first-coded diagnosis appeared in CPRD rather than HES (referred to as ‘diagnosed in primary care’);
- registered at current practice for at least 1 year pre-diagnosis;
- practices with consent to linkage to HES; and
- eligible for linkage with HES (England only), IMD data, and the ONS death register.

In a second analysis, we excluded patients with pre-existing asthma or HF.

### Measures of NICE guideline compliance

The recording of spirometry, chest X-ray, FBC, and calculation of BMI were each sought in the pre-diagnosis period. As a secondary analysis, we restricted this to the year before diagnosis. For assessment of variation between practices, spirometry recording was sought in the 6 months before and after diagnosis. This is the same definition used in the national audit^6^ and also accounts for the requirement of the QOF COPD indicator: diagnosis confirmed by spirometry between 3 months before and 6 months after diagnosis.^7^

### Statistical analysis

Variations between practices for each of the four measures were first summarised using descriptive statistics. Multilevel logistic regression models quantified the non-random variation between GP practices in spirometry within 6 months before and after diagnosis. First, we fitted a ‘null’ model with only the practice-level random intercept to calculate the intraclass correlation coefficient (ICC). The ICC indicates how much of the total variation in the outcome is explained by between-practice variation. Next, the number of patients with COPD per practice was added to the empty model to give adjusted odds ratio (ORs). The median odds ratio (MOR) was reported.^8^ The MOR is a median of the set of odds ratios that could be obtained by comparing two patients with identical characteristics from two randomly selected practices. Funnel plots with 95% (2 standard deviation [SD]) and 99.7% (3 SD) control limits were used to graphically present the variation between practices, plotting the proportion of patients having spirometry against the number of patients with COPD per practice. This model was run for both the whole cohort and for just those who were diagnosed in primary care, accounting for GPs being potentially unaware of the patient’s diagnosis after hospitalisation.

## Results

In total, cohort 1 had 40 577, cohort 2 had 48 249, and cohort 3 (COVID-19 era) had 4752 patients. For patients without pre-existing asthma or HF, these totals were 31 676, 37 393, and 3368, respectively.

Overall, the mean age was 68.3 years, with 46.1% female; there was little change in either variables across the cohorts (Table 1 for patients without pre-existing asthma or HF). Regarding smoking status, 85.2% were recorded as ex-smokers or current smokers. Comorbidity was common, especially hypertension (42.9% overall), depression (25.2%), and anxiety (21.4%) (see Supplementary Table S1 for all comorbidities).

**Table 1.** Patient characteristics

Variable		2006–2007, *n* = 31 676, *n* (%)^a^	2016–2017, *n* = 37 393, *n* (%)^a^	March–August 2020, *n* = 3368, *n* (%)^a^	Total, *n* (%)^a^
Sex	Female	14 583 (46.0)	17 196 (46.0)	1591 (47.2)	33 370 (46.1)
	Male	17 093 (54.0)	20 197 (54.0)	1777 (52.8)	39 069 (53.9)
Age, years	Mean (SD)	67.5 (11.3)	66.5 (11.4)	66.4 (11.7)	68.3 (12.0)
IMD quintile	1 (least deprived)	4692 (14.8)	5508 (14.7)	468 (13.9)	10 668 (14.7)
	2	5684 (17.9)	6544 (17.5)	559 (16.6)	12 787 (17.7)
	3	5968 (18.8)	6932 (18.5)	648 (19.2)	13 548 (18.7)
	4	6683 (21.1)	8181 (21.9)	762 (22.6)	15 626 (21.6)
	5 (most deprived)	8621 (27.2)	10 208 (27.3)	929 (27.6)	19 758 (27.3)
	Missing	28 (0.1)	20 (0.1)	<5 (0.1)	NA (0.1)
Smoking status	Missing	776 (2.4)	2129 (5.7)	115 (3.4)	3020 (4.2)
	Never smoker	3599 (11.4)	3734 (10.0)	349 (10.4)	7682 (10.6)
	Ex-smoker	12 306 (38.8)	13 978 (37.4)	1345 (39.9)	27 629 (38.1)
	Current smoker	14 995 (47.3)	17 552 (46.9)	1559 (46.3)	34 106 (47.1)
BMI measured	Yes	28 917 (91.3)	36 430 (97.4)	3272 (97.1)	68 619 (94.7)
	No	2759 (8.7)	963 (2.6)	96 (2.9)	3818 (5.3)
BMI category	Underweight (<18.5 kg/m^2^)	1413 (4.5)	1473 (3.9)	130 (3.9)	3016 (4.2)
	Normal (18.5–24.9 kg/m^2^)	10 905 (34.4)	12 185 (32.6)	1119 (33.2)	24 209 (33.4)
	Overweight (25.0–29.9 kg/m^2^)	9762 (30.8)	12 223 (32.7)	1010 (30.0)	22 995 (31.7)
	Obese (≥30 kg/m^2^)	6837 (21.6)	10 549 (28.2)	1013 (30.1)	18 399 (25.4)
	Missing	2759 (8.7)	963 (2.6)	96 (2.9)	3818 (5.3)
Current asthma	Yes	8170 (25.8)	7356 (19.7)	672 (20.0)	16 198 (22.4)
Congestive heart failure	Yes	2008 (6.3)	2383 (6.4)	286 (8.5)	4677 (6.5)
Total number of comorbidities	Mean (SD)	3.5 (1.9)	4.2 (2.1)	4.2 (2.3)	3.9 (2.2)
GOLD status	GOLD stage 1: ≥80%	2748 (8.7)	8516 (22.8)	848 (25.2)	12 112 (16.7)
	GOLD stage 2: 50%–79%	11 722 (37.0)	15 839 (42.4)	913 (27.1)	28 474 (39.3)
	GOLD stage 3: 30%–49%	4927 (15.6)	4066 (10.9)	217 (6.4)	9210 (12.7)
	GOLD stage 4:<30%	819 (2.6)	502 (1.3)	26 (0.8)	1347 (1.9)
	Missing FEV1 %-predicted measurement	11 460 (36.2)	8470 (22.7)	1364 (40.5)	21 294 (29.4)

^a^Unless otherwise stated. BMI = body mass index. FEV1 = forced expiratory volume. GOLD = Global Initiative for Chronic Obstructive Lung Disease. IMD = Index of Multiple Deprivation. NA = not available. SD = standard deviation.

Table 2 shows the compliance with NICE guidelines for diagnosing COPD for all patients; for patients without pre-existing asthma or HF, see Supplementary Table S2. The use of pre-diagnosis spirometry improved in cohort 2 (74.2%) on cohort 1 (62.8%), but fell back for the COVID-19 cohort (61.1%). In contrast, chest X-ray, FBC, and BMI all improved after cohort 1 and were maintained for the COVID-19 cohort, and almost all patients received one of these investigations, but the improvements were smaller when the analysis was restricted to 1 year before diagnosis. For example, for spirometry in the year before diagnosis, the proportions for cohorts 1, 2, and 3 were 55.7%, 63.8%, and 37.4%, respectively. The proportion receiving all four investigations before diagnosis jumped from 26.6% in cohort 1 to 46.7% in cohort 2 and was maintained in cohort 3 (43.0%). The proportions with all four investigations done were much smaller when restricted to the year before diagnosis.

**Table 2.** Compliance with NICE guidelines for COPD diagnosis, all patients

Variable		2006–2007, *n* (%)	2016–2017, *n* (%)	March–August 2020, *n* (%)	Total, *n* (%)	*P*-value
Spirometry (pre-diagnosis)	Yes	25 472 (62.8)	35 800 (74.2)	2902 (61.1)	64 174 (68.6)	<0.001
	No	15 105 (37.2)	12 449 (25.8)	1850 (38.9)	29 404 (31.4)	
CXR	Yes	19 859 (48.9)	31 138 (64.5)	3186 (67.0)	54 183 (57.9)	<0.001
	No	20 718 (51.1)	17 111 (35.5)	1566 (33.0)	39 395 (42.1)	
FBC	Yes	30 470 (75.1)	42 798 (88.7)	4381 (92.2)	77 649 (83.0)	<0.001
	No	10 107 (24.9)	5451 (11.3)	371 (7.8)	15 929 (17.0)	
BMI	Yes	36 060 (88.9)	46 321 (96.0)	4555 (95.9)	86 936 (92.9)	<0.001
	No	4517 (11.1)	1928 (4.0)	197 (4.1)	6642 (7.1)	
All of spirometry, FBC, CXR, and BMI measurement before COPD diagnosis	Yes	10 811 (26.6)	22 541 (46.7)	2043 (43.0)	35 395 (37.8)	<0.001
	No	29 766 (73.4)	25 708 (53.3)	2709 (57.0)	58 183 (62.2)	
At least one of FBC, CXR, and BMI measurement before COPD diagnosis	Yes	39 055 (96.2)	47 536 (98.5)	4684 (98.6)	91 275 (97.5)	<0.001
	No	1522 (3.8)	713 (1.5)	68 (1.4)	2303 (2.5)	
Spirometry, CXR, BMI, and FBC	Spirometry and one of CXR, BMI, or FBC	25 128 (61.9)	35 719 (74.0)	2900 (61.0)	63 747 (68.1)	<0.001
	Spirometry only	344 (0.8)	81 (0.2)	2 (0.04)	427 (0.5)	
	No spirometry and one of CXR, BMI, or FBC	13 927 (34.3)	11 817 (24.5)	1784 (37.5)	27 528 (29.4)	
	No spirometry, CXR, BMI, or FBC	1178 (2.9)	632 (1.3)	66 (1.4)	1876 (2.0)	
Spirometry in the year before COPD diagnosis	Yes	23 130 (57.0)	29 800 (61.8)	1657 (34.9)	54 587 (58.3)	<0.001
	No	17 447 (43.0)	18 449 (38.2)	3095 (65.1)	38 991 (41.7)	
CXR in the year before COPD diagnosis	Yes	9978 (24.6)	16 345 (33.9)	1513 (31.8)	27 836 (29.7)	<0.001
	No	30 599 (75.4)	31 904 (66.1)	3239 (68.2)	65 742 (70.3)	
FBC in the year before COPD diagnosis	Yes	19 556 (48.2)	27 222 (56.4)	2666 (56.1)	49 444 (52.8)	<0.001
	No	21 021 (51.8)	21 027 (43.6)	2086 (43.9)	44 134 (47.2)	
BMI measurement in the year before COPD diagnosis	Yes	22 917 (56.5)	29 158 (60.4)	2595 (54.6)	54 670 (58.4)	<0.001
	No	17 660 (43.5)	19 091 (39.6)	2157 (45.4)	38 908 (41.6)	
All of spirometry, FBC, CXR, and BMI measurement in the year before COPD diagnosis	Yes	2807 (6.9)	5598 (11.6)	366 (7.7)	8771 (9.4)	<0.001
	No	37 770 (93.1)	42 651 (88.4)	4386 (92.3)	84 807 (90.6)	
At least one of FBC, CXR, and BMI measurement in the year before COPD diagnosis	Yes	31 892 (78.6)	41 027 (85.0)	3858 (81.2)	76 777 (82.0)	<0.001
	No	8685 (21.4)	7222 (15.0)	894 (18.8)	16 801 (18.0)	
	Spirometry and one of CXR, BMI, or FBC	19 943 (49.1)	27 257 (56.5)	1520 (32.0)	48 720 (52.1)	
	Spirometry only	3187 (7.9)	2543 (5.3)	137 (2.9)	5867 (6.3)	
	No spirometry and one of CXR, BMI, or FBC	11 949 (29.4)	13 770 (28.5)	2338 (49.2)	28 057 (30.0)	
	No spirometry, CXR, BMI, or FBC	5498 (13.5)	4679 (9.7)	757 (15.9)	10 934 (11.7)	

BMI = body mass index. COPD = chronic obstructive pulmonary disease. CXR = chest X-ray. FBC = full blood count. NICE = National Institute for Health and Care Excellence.

### Variation between practices

Table 3 shows the variation by practice in NICE guideline compliance for those diagnosed in primary care; Supplementary Table S3 reports the figures for all patients combined.

**Table 3.** Between-practice variation in compliance with NICE guidelines for COPD diagnosis

Variable	2006–2007, median % (IQR)	2016–2017, median % (IQR)	March–August 2020, median % (IQR)	All cohorts, median % (IQR)
Spirometry (pre-diagnosis)	76.9 (61.2–86.6)	86.4 (78.6–93.3)	80.0 (50.0–100.0)	80.7 (72.3–86.7)
CXR	50.0 (27.3–71.0)	73.1 (50.0–85.7)	80.0 (50.0–100.0)	61.5 (43.2–75.6)
FBC	80.0 (66.7–89.3)	94.4 (86.7–100.0)	100.0 (100.0–100.0)	87.0 (79.4–92.6)
BMI	95.0 (87.5–100.0)	100.0 (96.7–100.0)	100.0 (100.0–100.0)	96.7 (92.6–100.0)
All of spirometry, FBC, CXR, and BMI measurement before COPD diagnosis	27.8 (11.8–44.4)	54.9 (33.3–69.0)	50.0 (0.0–80.0)	42.9 (27.8–55.6)
At least one of FBC, CXR, and BMI measurement before COPD diagnosis	100.0 (97.4–100.0)	100.0 (100.0–100.0)	100.0 (100.0–100.0)	100.0 (98.4–100.0)
None of spirometry, FBC, CXR, or BMI measurement	0.0 (0.0–0.0)	0.0 (0.0–0.0)	0.0 (0.0–0.0)	0.0 (0.0–0.0)
Spirometry in the year before COPD diagnosis	70.8 (55.0–82.4)	77.8 (66.7–86.4)	50.0 (0.0–78.4)	72.6 (63.5–80.0)
CXR in the year before COPD diagnosis	25.0 (7.7–41.7)	39.2 (10.0–58.2)	25.0 (0.0–57.1)	32.4 (15.9–45.5)
FBC in the year before COPD diagnosis	50.0 (37.5–61.9)	62.5 (47.8–73.1)	60.0 (3.1–100.0)	56.0 (43.8–65.0)
BMI measurement in the year before COPD diagnosis	61.5 (46.2–76.7)	66.8 (51.3–80.0)	60.0 (33.3–100.0)	64.1 (52.6–74.2)
All of spirometry, FBC, CXR, and BMI measurement in the year before COPD diagnosis	4.4 (0.0–12.8)	8.7 (0.0–23.1)	0.0 (0.0–0.0)	9.1 (3.0–17.4)
At least one of FBC, CXR, and BMI measurement in the year before COPD diagnosis	83.8 (75.0–92.9)	90.2 (82.9–96.6)	100.0 (71.4–100.0)	86.4 (80.0–92.0)
None of spirometry, FBC, CXR, or BMI measurement in the year before COPD diagnosis	5.9 (0.0–12.9)	3.3 (0.0–8.2)	0.0 (0.0–16.7)	5.9 (2.7–10.0)

BMI = body mass index. COPD = chronic obstructive pulmonary disease. CXR = chest X-ray. FBC = full blood count. IQR = interquartile range. NICE = National Institute for Health and Care Excellence.

**Figure 1.**
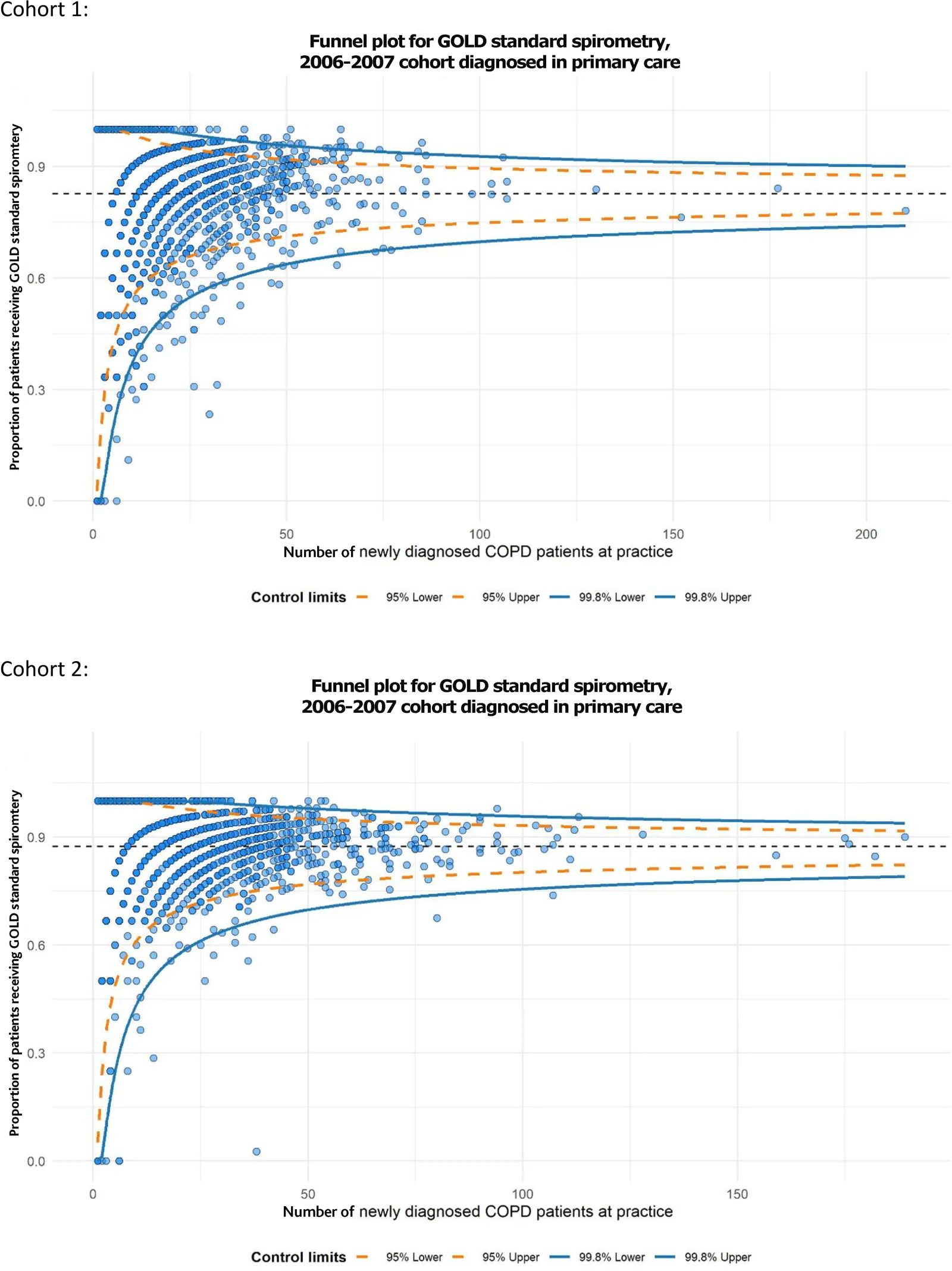
Funnel plot of proportion of patients following the initial NICE pathways (performed spirometry within 6 months before and after COPD diagnosis) by number of COPD patients per practice. COPD = chronic obstructive pulmonary disease. GOLD = Global Initiative for Chronic Obstructive Lung Disease. NICE = National Institute for Health and Care Excellence.

The ICC and MORs for spirometry are shown in Table 4. There was most non-random variation between practices for cohort 1, but the MORs show considerable variation by practice for all three cohorts. At least 20% of practices were outliers on funnel plots at 2 SD (Figure 1 and Table 5). With purely random variation, we would expect 5% of practices to be outliers at 2 SD and just 0.3% at 3 SD. The number of patients with COPD at the practice was significantly and positively associated with spirometry for cohort 1 only, but the size of the effect was negligible.

**Table 4.** Results of multilevel analysis for use of spirometry around the time of COPD diagnosis

Statistic	2006–2007 cohort	2016–2017 cohort	March–August 2020 cohort
*n*	31 676	37 393	3368
Median *n* per practice (IQR)	18 (9–31)	21 (11–35)	2 (1–4)
ICC: null model	0.13	0.08	0.09
Median OR: null model (bootstrap 95% CI)	1.95 (1.89 to 1.98)	1.62 (1.59 to 1.65)	1.72 (1.68 to 1.76)
ICC: COPD patient number as predictor	0.12	0.08	0.09
Median OR: COPD patient number as predictor (bootstrap 95% CI)	1.92 (1.87 to 1.96)	1.67 (1.63 to 1.69)	1.72 (1.68 to 1.76)
OR (number of COPD patients at practice) (95% CI)	1.01 (1.00 to 1.01)	1.00 (1.00 to 1.00)	1.01 (0.98 to 1.03)
*P*-value (number of COPD patients at practice)	<0.001	0.097	0.605

COPD = chronic obstructive pulmonary disease. ICC = intraclass correlation coefficient. IQR = interquartile range. OR = odds ratio.

**Table 5.** Funnel plot outlier practices for spirometry within 6 months before and after COPD diagnosis by cohort

Outlier status	2006–2007 cohort, *n* (%)	2016–2017 cohort, *n* (%)
Not an outlier	1057 (75.5)	1144 (80.3)
Over 3 SD above the average	61 (4.4)	47 (3.3)
Between 2 and 3 SD above the average	148 (10.6)	154 (10.8)
Between 2 and 3 SD below the average	92 (6.6)	56 (3.9)
More than 3 SD below the average	42 (3.0)	23 (1.6)
Total number of practices	1400 (100)	1424 (100)

COPD = chronic obstructive pulmonary disease. SD = standard deviation.

Given the small number of patients per practice in cohort 3, we only show the funnel plot for the first two cohorts.

## Discussion

### Summary

Overall, approximately two in three patients received pre-diagnosis spirometry. The proportion improved in cohort 2 on cohort 1 but fell back for the early COVID-19-era cohort. In contrast, chest X-ray, FBC, and BMI all improved after cohort 1 and were maintained for the COVID-19-era cohort. Almost all patients received one of these recommended tests and measurements, but only a small minority received all four. We found much non-random variation in spirometry use between practices.

### Strengths and limitations

CPRD is much used in research. Its main strengths include relatively easy access, large sample size, broad representativeness of the UK population, and the almost universal coverage by primary care in the UK. Its longevity means that many groups have experience with it and have conducted validation studies on the accuracy of disease and date coding. The QOF incentivises the recording of chronic diseases, including COPD, GP actions that are important for diagnosis, and follow-up such as spirometry and blood tests, and key risk factors such as BMI and smoking.

Our COVID-19-era cohort was fairly small. As with the other two cohorts, it was defined according to the date of diagnosis (first recording) of COPD. This means that many patients would have had their tests before the pandemic onset, so it does not fully reflect the situation during the pandemic. Owing to subsequent COVID-19 disruptions of the health service, there may have been more misdiagnosis for this cohort than for the earlier two.

While CPRD codes for COPD may be mostly accurate, misdiagnosis for the condition is a well-known problem. A 2018 review suggested that approximately 70% of COPD worldwide may be underdiagnosed, whereas between 30% and 60% of patients with a previous physician diagnosis of COPD may not actually have the disease and have therefore been overdiagnosed.^9^ Requesting the NICE-recommended tests is one key element to diagnosis. However, we did not investigate the GPs’ ability to correctly interpret those tests. Previously, our group found that the GPs correctly spotted obstruction in only three in four cases, less often if the patient had asthma.^10^

### Comparison with existing literature

The most direct comparison with our study is the national audit, although it relates to practices in Wales rather than in England and did not assess between-practice variation, as in our study. The most recent report^6^ is based on patients coded with the disease between April 2020 and July 2021, which subsumes the timeframe for our cohort 3 and extends it further into the COVID-19 era. Its key findings for COPD relevant here are that just 1.9% had post-bronchodilator spirometry in the previous 2 years, and, overall, no improvement since the previous audit. We were unable to assess specifically post-bronchodilator spirometry but also found low levels of this test overall. Spirometry use in other countries has also been found to be low. A US study of 101 patients from two community clinics found a low rate (21%) of lung function testing.^11^ In a German primary care network, record-keeping was not standardised and spirometry in the year before diagnosis was just 29%.^12^ In an Italian study of 300 patients sent for spirometry, of whom only 75 had COPD, the authors made a link between the low use of spirometry to help diagnose asthma and COPD and the low proportion of doctor-diagnosed COPD with their concordant spirometry findings.^13^

QOF has facilitated studies on the association between the quality and organisation of primary care and outcomes for long-term conditions. For COPD, these studies cover aspects of quality of care other than the four NICE-recommended tests that we investigated. A London study using data from 2006–2010 used these QOF indicators for COPD: proportion of patients who had received the influenza vaccination in the preceding 12 months; confirmation of diagnosis using spirometry; recording of FEV1 in the previous 15 months; recording of good inhaler technique; and patients who had had a review by a health professional in the preceding 15 months. Emergency hospital admission rates were found to be related to COPD prevalence and small-area deprivation but not to GP QOF performance, GP supply, or nurse supply.^14^ Another QOF study looked at the influence of primary care nursing levels on QOF performance in chronic disease management, including that for COPD. This made use not only of the usual clinical indicators but also those describing organisational performance. The many organisational variables were reduced by factor analysis. Although the relation with staffing levels was statistically significant, the strongest predictors of quality of care were found to be the organisational factors of clinical recording, education and training, and use of patient experience surveys.^15^ An interaction term suggested that the adverse effects on quality of the lowest levels of nurse staffing were mitigated by good organisational quality.

The between-practice variation in NICE guideline compliance for COPD is in line with our previous CPRD analysis for patients with HF in which we focused on following the steps of serum natriuretic peptide test and/or echocardiogram, and referral to a specialist; a timeframe of 6 months since presentation with a HF symptom was used. In that study, 13% of practices were identified as 3 SD funnel plot outliers, with an ICC of 8.6% (4.9% following adjustment for patient factors).^16^

A Danish study on 2008 data for *‘patients over 18 years receiving first-time prescriptions for medication targeting obstructive lung disease’* found that practices with partnerships were more likely than single-handed, and older GPs less likely, to order spirometry; practices with training status were more likely to order spirometry.^17^ Unfortunately, this level of detail on practices is not available within CPRD.

### Implications for research and practice

A 2010 King’s Fund review of the evidence of the quality of diagnosis and referral in UK primary care noted the challenges facing GPs such as vague symptoms, overlap in symptoms for serious common conditions, and the modest predictive value of diagnostic tests in primary care. These are complicated by limited consultation times, differences in GP knowledge, skills, and attitudes, access to diagnostics, and the time taken to receive test results.^18^ To mitigate these challenges, the 2019 *NHS Long Term Plan* set out plans for more joined-up and coordinated care, including the creation of primary care networks that would work together to deliver services such as spirometry. Earlier diagnosis would lead to early interventions for smoking cessation, better management, and improved health outcomes.

Access to spirometry is clearly improved when the kit is present in the practice, but ownership of a spirometer was already 60%–80% in the UK by 2005.^19,20^ The use of spirometry in primary care stopped during the early COVID-19 waves as spirometry-associated cough can generate aerosol droplets, but it has resumed with published guidance on restarting it with risk mitigation.^21^

The shift towards remote consultations in primary care, which has persisted post-pandemic, may have implications for the investigation and diagnosis of a condition where an important element of assessment is physical examination, although improved access through online consultations may result in earlier investigation. Unfortunately, whether the consultations are remote or face to face is currently poorly coded and not reliably identifiable in routinely collected primary care datasets such as CPRD. In general, it is important that coding is consistent and accurate, as high-quality patient records are the foundation of good clinical care delivery and not just useful for research.

In conclusion, the use of NICE-recommended diagnosis tests, particularly spirometry and chest X-rays, warrants further improvement. The COVID-19 pandemic hit many diagnostic and preventive services hard, and it will be important to monitor these effects; our analysis can serve as baseline data for this monitoring.

## Data

The anonymised patient data that were used for this study can be accessed by contacting the CPRD at enquiries@cprd.com. Access to these data is subject to a data sharing agreement containing detailed terms and conditions of use following protocol approval from CPRD’s research data governance process via the eRAP portal.
